# MegaPlantTF: a machine learning framework for comprehensive identification and classification of plant transcription factors

**DOI:** 10.1093/bioinformatics/btaf678

**Published:** 2025-12-23

**Authors:** Genereux Akotenou, Asmaa H Hassan, Morad M Mokhtar, Achraf El Allali

**Affiliations:** Bioinformatics Laboratory, College of Computing, University Mohammed VI Polytechnic, Ben Guerir 43150, Morocco; Bioinformatics Laboratory, College of Computing, University Mohammed VI Polytechnic, Ben Guerir 43150, Morocco; Chemical and Biochemical Sciences—Green Process Engineering, University Mohammed VI Polytechnic, Ben Guerir 43150, Morocco; Bioinformatics Laboratory, College of Computing, University Mohammed VI Polytechnic, Ben Guerir 43150, Morocco

## Abstract

**Motivation:**

Understanding the role of transcription factors (TFs) in plants is essential for the study of gene regulation and various biological processes. However, both TF detection and classification remain challenging due to the great diversity and complexity of these proteins. Conventional approaches, such as BLAST, often suffer from high computational complexity and limited performance on less common TF families.

**Results:**

We introduce MegaPlantTF, the first comprehensive machine learning and deep learning framework for the prediction (TF versus non-TF) and classification (family-level) of plant TFs. Our method employs *k*-mer-based protein representations and a two-stage architecture combining a deep feed-forward neural network with a stacking ensemble classifier. To ensure robust performance assessment, we report micro-, macro-, and weighted-average performance metrics, providing a holistic evaluation of both frequent and underrepresented TF families. Additionally, we employ threshold-based evaluation to calibrate confidence in TF detection. The results show that MegaPlantTF achieves strong accuracy and precision, particularly with a *k*-mer size of 3 and a classification threshold of 0.5, and maintains stable performance even under stringent thresholds. In addition to the standard cross-validation tests, a use case study on *Sorghum bicolor* confirms that our method performs strongly in the genome-wide analysis, making it highly suitable for large-scale TF identification and classification tasks. MegaPlantTF represents a novel contribution by integrating *k*-mer encoding, binary family-specific classifiers, and a two-stage stacking ensemble into a unified, reproducible framework for large-scale plant TF identification and classification.

**Availability and implementation:**

MegaPlantTF is freely accessible through a public web server available at https://bioinformatics.um6p.ma/MegaPlantTF. The complete source code, including pretrained models and example datasets, is available at https://github.com/Bioinformatics-UM6P/MegaPlantTF.

## 1 Introduction

Transcription factors (TFs) are important regulatory proteins in plants that control gene expression by binding to specific DNA sequences (Yang et al. 2004). TFs are modular proteins consisting of different domains, which include DNA-binding domains that recognize specific DNA sequences, transcriptional regulatory domains that modulate gene expression, oligomerization sites for protein–protein interactions, and nuclear localization signals for targeting TFs to the nucleus. TFs bind to cis-elements within gene promoters and influence the transcription of target genes (Hong 2016, Chowdhary et al. 2023) and play a crucial role in plant biology. They regulate various developmental processes, including embryogenesis and seed development (Zhang *et al.* 2022, Hussain *et al.* 2024), as well as the response to biotic and abiotic stresses (Zhou *et al.* 2017, Wang *et al.* 2023). In addition, TFs are involved in secondary metabolism, which is important for the production of compounds that aid in defense and adaptation of plants (Thakur and Vasudev 2022). TFs regulate gene expression through a complex interplay of mechanisms. They bind to specific DNA sequences on gene promoters and activate or repress transcription (López-González *et al.* 2019). In addition, they are involved in protein–protein interactions (Alves *et al.* 2014) and post-translational modifications such as phosphorylation affect the activity, stability, and localization of TFs, which ultimately influence the plant’s response to various environmental stresses (Li and Loake 2016, Baoxiang *et al.* 2023).

There are different groups of TFs in plants, which are classified according to their structural characteristics and functions. The most common are WRKY, NAC, AP2/ERF, and MYB (Chowdhary *et al.* 2023). There are several databases and tools that provide comprehensive resources and recognize TFs in plants. The Plant Transcription Factor Database (PlnTFDB) provides a comprehensive collection of TFs and transcriptional regulators for 19 plant species and allows cross-species comparisons (Pérez-Rodríguez *et al.* 2010). Currently, there are few computational tools dedicated to the classification of plant TFs. For example, PlantTFcat identifies and categorizes TF genes based on domain patterns (Dai *et al.* 2013). This tool limits the input size to a maximum of 200 MB, making it unsuitable for genome-wide TF analysis. The PlnTFDB platform also provides a TF classification tool that integrates multiple prediction methods to systematically identify and annotate plant TFs (Chang *et al.* 2008). Additionally, iTAK (Zheng *et al.* 2016) detects TFs based on conserved domains and classification rules specific to plants. PANTHER, although not plant-specific, further classifies these TFs into functional categories such as DNA-binding proteins, transcription regulators, and signal transduction factors (Thomas *et al.* 2003). Together, these resources provide valuable platforms for the systematic identification and classification of plant TFs; however, expanding their accuracy, scalability and coverage could further enhance their utility for genome-wide studies.

Advances in plant genomics have highlighted the need for efficient and accurate methods to detect TFs in the genome. Traditional approaches have relied predominantly on computational or similarity-based techniques, such as motif discovery and sequence alignment, which can be resource-intensive and less practical for large-scale genomic studies. In this context, machine learning offers a valuable alternative that can automatically recognize TFs in the plant genome and classify them into their respective groups with improved accuracy and efficiency. This approach reduces the computational burden and improves the scalability of TF classification tasks. In this study, we propose the first machine learning approach to optimize the detection and classification of TFs in plant genomes. Our main contributions are: (i) the construction of balanced, family-specific subsets derived from the PlantTFDB v4.0 dataset to enable robust binary classification, (ii) a systematic evaluation of *k*-mer–based encoding and feature selection strategies for protein sequence representation, (iii) the integration of a two-stage neural ensemble model combining binary classifiers with a stacking meta-classifier for improved prediction accuracy, and (iv) the development of an open-source, reproducible software tool (MegaPlantTF) that allows users to perform large-scale TF prediction and classification analyses efficiently from their local cluster as well as from the online web server.

## 2 Materials and methods

### 2.1 Dataset

The data used to build the models in this study are available from the joint program “PlantRegMap/PlantTFDB v4.0” by the Center for Bioinformatics at Peking University. PlantRegMap provides comprehensive functional regulatory maps in plants by integrating TFs and their regulatory interactions across various plant species (Tian *et al.* 2020) while PlantTFDB 4.0 provides a central repository for plant TFs and regulatory information (Jin *et al.* 2017). The construction of PlantTFDB was based on large-scale aggregation of plant proteomes from species with assembled genomes, complemented by translated transcript assemblies (“Plant Unique Transcripts”, PUTs) for species lacking complete genomes. These PUTs were derived from mRNA and EST data available in PlantGDB and translated into protein sequences using FrameFinder. Putative TFs were then systematically identified through HMMER searches against Pfam hidden Markov models (HMMs) corresponding to known DNA-binding domains (DBDs). For families lacking Pfam profiles, the authors generated custom HMMs from manually curated multiple sequence alignments of representative members, trimmed to conserved DBD regions, and built using hmmbuild (Finn *et al.* 2006). In early releases, TFs were identified using a global *E*-value cutoff (1 × 10${}^{-2}$) and domain-combination rules to resolve multi-domain cases. Orthologous relationships were initially established via all-against-all BLAST using a BLAST Score Ratio (BSR) threshold of 0.4. Subsequent releases refined these pipelines by incorporating non-redundant reference proteomes (integrating RefSeq, UniGene, and PlantGDB data), upgrading to HMMER3 with domain-specific bit-score thresholds, and applying rule-based domain filters (auxiliary and forbidden domains) to improve classification accuracy. Continuous manual curation was applied to validate family definitions, domain boundaries, and ambiguous assignments. Annotations were further expanded with InterProScan functional domains, gene ontology (GO) terms, expression and regulatory data, and orthology predictions using TribeMCL, OrthoMCL, and later OrthoFinder with reciprocal best hits. By versions 3.0–4.0, the database had scaled from 26,000 TFs across 22 species to over 320 000 TFs spanning more than 150 plant species. The latest release also integrates curated TF-binding motifs, genome-wide regulatory elements (e.g. ChIP-seq peaks, DNase footprints), and the companion PlantRegMap portal for TF prediction, motif scanning, and functional enrichment analyses (Jin *et al.* 2017).

**Figure 1.**
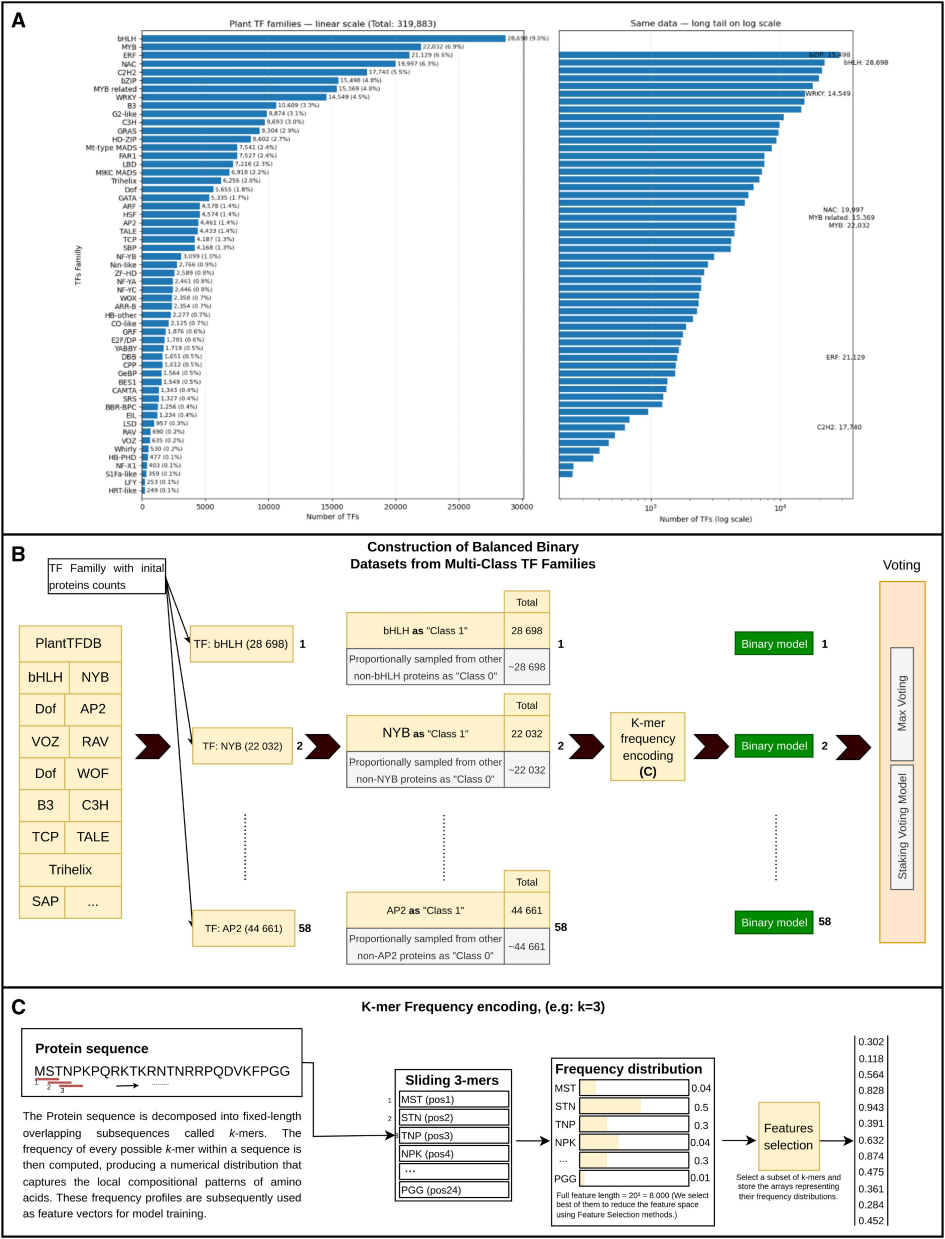
Dataset construction and feature encoding for plant transcription factor classification: (A) Distribution of transcription factor (TF) families from PlantTFDB v4.0. The left panel shows the total number of TFs per-family on a linear scale, highlighting the most abundant classes such as *bHLH*, *MYB*, *ERF*, and *NAC*. The right panel presents the same data on a logarithmic scale to illustrate the long-tail distribution across 58 TF families, where a small number of dominant families account for most TFs while many smaller families remain underrepresented. (B) Construction of balanced binary datasets from multi-class TF families for family-specific model training. (C) Illustration of *k*-mer frequency encoding used to convert protein sequences into numerical feature vectors.

In this work, we used all available TF protein sequences provided in PlantTFDB v4.0, totaling 320 370 TFs as of 23 May 2024. No families or species were excluded from the dataset. Only minimal preprocessing was applied to ensure consistency, including removal of exact duplicate entries and verification of valid amino acid characters. No additional filtering, alignment, or manual wrangling was required since PlantTFDB provides high-quality, curated protein sequences, and family annotations. These sequences were directly used to generate *k*-mer representations and model features as described in the following sections. The complete dataset can be accessed at planttfdb.gao-lab.org. Figure 1A shows the distribution of TF families in the dataset, highlighting the major and minor TF classes, while a detailed summary listing all 58 TF families and their corresponding sequence counts is provided in Table S1, available as supplementary data at *Bioinformatics* online.

**Table 1.** Evaluation metrics comparison between BLAST, max voting, and two-stage approaches for different thresholds.^a^

			Max voting												Two-stage											
	Metrics	BLAST	Threshold = 0.0				Threshold = 0.5				Threshold = 0.95				Threshold = 0.0				Threshold = 0.5				Threshold = 0.95			
			*k* = 2	*k* = 3	*k* = 4	*k* = 5	*k* = 2	*k* = 3	*k* = 4	*k* = 5	*k* = 2	*k* = 3	*k* = 4	*k* = 5	*k* = 2	*k* = 3	*k* = 4	*k* = 5	*k* = 2	*k* = 3	*k* = 4	*k* = 5	*k* = 2	*k* = 3	*k* = 4	*k* = 5
	Accuracy	0.853	0.954	0.930	0.986	0.958	0.952	0.914	0.977	0.954	0.929	0.852	0.948	0.934	0.981	**0.993**	0.992	0.972	0.979	0.993	0.991	0.963	0.946	0.980	0.976	0.947
	Precision	0.853	0.954	0.930	0.986	0.958	0.952	0.914	0.977	0.954	0.929	0.852	0.948	0.934	0.981	**0.993**	0.992	0.972	0.979	0.993	0.991	0.963	0.946	0.980	0.976	0.947
Micro	Recall	0.853	0.954	0.930	0.986	0.958	0.952	0.914	0.977	0.954	0.929	0.852	0.948	0.934	0.981	**0.993**	0.992	0.972	0.979	0.993	0.991	0.963	0.946	0.980	0.976	0.947
	*F*1	0.853	0.954	0.930	0.986	0.958	0.952	0.914	0.977	0.954	0.929	0.852	0.948	0.934	0.981	**0.993**	0.992	0.972	0.979	0.993	0.991	0.963	0.946	0.980	0.976	0.947
Macro	Precision	0.963	0.891	0.971	0.979	0.977	0.895	0.972	0.978	0.978	0.940	0.974	0.978	0.978	0.972	0.992	**0.995**	0.991	0.961	0.978	0.979	0.979	0.977	0.981	0.981	0.982
	Recall	0.854	0.951	0.951	0.990	0.972	0.950	0.938	0.984	0.969	0.929	0.869	0.960	0.939	0.971	**0.992**	0.992	0.978	0.971	0.992	0.991	0.975	0.921	0.982	0.977	0.960
	*F*1	0.894	0.911	0.949	0.980	0.966	0.908	0.941	0.972	0.964	0.922	0.885	0.960	0.945	0.971	0.992	**0.993**	0.983	0.957	0.976	0.976	0.968	0.937	0.973	0.970	0.961
Weighted	Precision	0.975	0.958	0.991	0.988	0.995	0.961	0.993	0.996	0.996	0.973	0.997	0.998	0.997	0.981	0.993	0.993	0.979	0.984	0.994	0.995	0.996	0.995	0.998	0.999	**0.999**
	Recall	0.853	0.954	0.930	0.986	0.958	0.952	0.914	0.977	0.954	0.929	0.852	0.948	0.934	0.981	**0.993**	0.992	0.972	0.979	0.993	0.991	0.963	0.946	0.980	0.976	0.947
	*F*1	0.909	0.955	0.958	0.986	0.976	0.956	0.949	0.986	0.974	0.950	0.909	0.971	0.963	0.981	0.993	0.992	0.974	0.981	**0.994**	0.993	0.979	0.969	0.989	0.987	0.971

a Bold values indicate the best performance achieved for a given evaluation metric across all methods, thresholds, and k-mer sizes.

### 2.2 Data balancing

The class distribution as shown in Fig. 1A reveals a significant imbalance, with some classes being heavily underrepresented. In such cases, there are two common strategies to address this imbalance: *over-sampling* or *under-sampling*. However, both approaches have drawbacks. Over-sampling introduces duplicated sequences into the dataset, which generally does not improve model performance, while under-sampling leads to substantial information loss, as the smallest class contains only 109 sequences, compared to 28 698 in the largest class.

To overcome this challenge, we have implemented a binary classification strategy that uses small, balanced training subsets. This method avoids the pitfalls of over-sampling, which could introduce redundant or artificial sequences, and under-sampling, which could lead to the loss of valuable data. Our approach trains robust binary classifiers on carefully selected parts of the dataset and then combines their predictions for the final classification. To create balanced training subsets, we created a specific subset for each TF family, in which sequences from that family are labeled as class 1. Sequences from other families were proportionally sampled from the original dataset and labeled as class 0. This approach ensures a balanced class representation during the training of each binary classifier for the respective protein class. The workflow for transforming the multi-class TF dataset into balanced per-family binary subsets is depicted in Fig. 1B.

### 2.3 Feature extraction

Because machine learning models require numerical input, the amino acid sequences were transformed into quantitative representations suitable for computational modeling. To achieve this, we apply the “*k*-mer encoding,” in which the sequences are broken down into fixed-length subsequences called *k*-mers. This method transforms each sequence into a numerical vector by computing the *k*-mer frequencies, thus capturing local sequence patterns that are crucial for model training. Several *k*-mer sizes (k=2–5) were compared to identify the most informative representation. For k=2 the feature space consists of $20^{2}=400$ features. As *k* increases, the feature space grows exponentially, so that for k=3 it comprises 20^3^ = 8000 features, for k=4 it comprises 20^4^ = 160 000 features and for k=5 it comprises 20^5^ = 3 200 000 features. This exponential increase is due to the growing diversity of *k*-mers generated from the 20 standard amino acids. To illustrate this process, consider a short protein fragment such as MSTNPKPQRKTKRNTNRRPQDVKFPGG. For k=3, this sequence produces 26 overlapping 3-mers (e.g. MST, STN, TNP, NPK, PKP, …). The relative frequency of each unique 3-mer is computed and stored as an entry in the feature vector, resulting in an 8000-dimensional numerical embedding as depicted in Fig. 1C. These vectors are used as numerical inputs for training the classification models. Consequently, the selection of the optimal *k*-mer size, as well as the most representative features is crucial as it represents a balance between model complexity and computational feasibility.

### 2.4 Feature selection

Feature selection aims to reduce the dimensionality of the feature space while maintaining or improving the performance of the model. In this work, we use analysis of variance (ANOVA) as the primary feature selection method to refine the *k*-mer representations. ANOVA effectively identifies the *k*-mers that contribute significantly to the variability within the dataset, optimizing the efficiency of model training and prediction. We also explored alternative feature selection techniques, including *F*-tests, random forests (RF), chi-squared tests, LASSO (*L*1 regularization), and minimum redundancy maximum relevance (mRMR). Each method was applied to representative sample datasets derived from the *k*-mer–based feature matrices. After comparative evaluation, ANOVA proved to be the most effective for our purposes. Applying ANOVA, substantially reduced the dimensionality of the initial feature space^1^ while retaining the most informative features to facilitate training and decision-making for TF classification tasks. For k=2, we selected the 200 best features from the original 400. Similarly, for k=3,4,5, we kept the best 1000 features, starting from the original 8000, 160 000, and 3 200 000 features, respectively.

### 2.5 Machine learning model

To classify protein sequences into their respective TF families, we adopted a two-stage modeling strategy. In the first stage, a binary classifier was trained for each of the 58 TF families, and in the second stage, the predictions of these classifiers were aggregated to produce the final family assignment.

#### 2.5.1 Binary classifier

**Figure 2.**
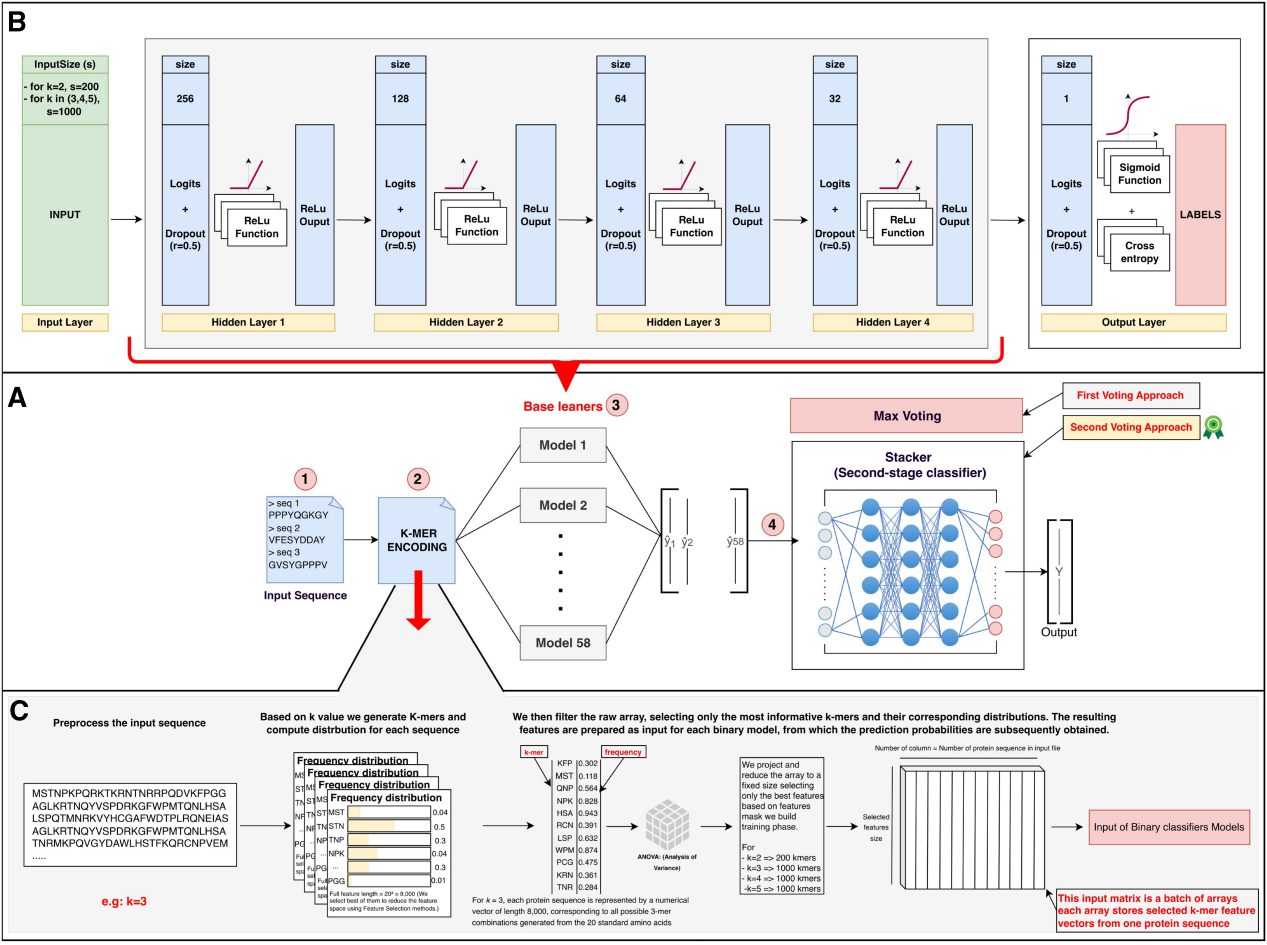
Overview of the MegaPlantTF workflow and binary classifier architecture. (A) Overall model pipeline showing the transformation of transcription factor (TF) protein sequences into numerical features through *k*-mer encoding. Each sequence is converted into frequency-based *k*-mer feature vectors and passed to 58 family-specific base learners (binary classifiers). Two ensemble strategies are used: a first-stage max-voting approach and a second-stage stacker meta-classifier for refined predictions. (B) Architecture of an individual binary feed-forward classifier. The network consists of four fully connected hidden layers (256, 128, 64, 32 neurons), each followed by ReLU activation and 0.5 dropout. The output layer uses a sigmoid activation to produce binary probabilities, trained with cross-entropy loss. (C) Feature preprocessing and selection workflow. Protein sequences are decomposed into overlapping *k*-mers, their frequency distributions are computed, and statistical filtering is applied via ANOVA to retain the most informative features. The resulting reduced feature matrices serve as standardized inputs for each binary classifier in the ensemble.

For heuristic evaluation, we compared the performance of several classifiers, including KNeighborsClassifier, XGB Classifier, Gaussian Process Classifier, Random Forest Classifier, Ada Boost Classifier, Gaussian NB, SVC with linear and radial basis function kernel, and a feed-forward neural network architecture. Among these, the feed-forward neural network achieved the best overall performance. The network architecture consists of an input layer corresponding to the reduced feature space of each *k*-mer size, followed by four fully connected (dense) layers with progressively decreasing neuron counts. Each layer employs ReLU activation to introduce nonlinearity, along with dropout regularization to prevent overfitting. The output layer uses a sigmoid activation to produce class probabilities for binary classification. The model was trained using binary cross-entropy loss and the Adam optimizer. An overview of the architecture is shown in Fig. 2B.

The preprocessing and feature selection steps used to generate the inputs for these models are illustrated in Fig. 2C. In this step, each protein sequence is decomposed into overlapping *k*-mers, from which frequency distributions are computed. The resulting frequency arrays are statistically filtered using ANOVA to retain only the most informative *k*-mers. These selected *k*-mers are then organized into fixed-length numerical vectors representing each protein sequence. To prepare the data for neural network input, these vectors are arranged into a two-dimensional matrix where each row corresponds to a distinct protein sequence and each column represents a selected *k*-mer feature. Thus, the input tensor has the shape (number of sequences × number of selected *k*-mers). For example, for k=3 and 1000 selected features, a batch of 50 protein sequences is represented as a (50×1000) input matrix. This matrix is then fed into the neural network as a batch tensor of standardized numerical features. The same procedure is applied for all *k*-mer sizes, producing consistent input tensors that serve as the standardized representation for training the binary classifiers.

#### 2.5.2 Final classifier

For each input sequence, the output probabilities produced by the 58 binary classifiers are combined to generate the final prediction using two complementary ensemble strategies: max voting and stacking (second-stage classifier). Max voting provides a straightforward consensus mechanism that selects the class with the highest predicted probability among all base classifiers. Stacking (second-stage classifier) is designed to further enhance prediction robustness and accuracy. In this approach, the probability outputs from the 58 binary classifiers are used as input features for a meta-classifier (the stacking model), which learns how to combine these probabilities optimally to yield refined final class predictions. As shown in Fig. 2A, the stacking model follows the same feed-forward architecture as the individual binary classifiers (see Fig. 2B), differing only in its input and output dimensions. Separate models were trained for each *k*-mer size (k=2,3,4,5), using an 80/20 train–test split for performance evaluation. The complete MegaPlantTF workflow is illustrated in Fig. 2, beginning with input sequence acquisition, followed by *k*-mer encoding (detailed in subplot C), inference through 58 binary classifiers, and a final voting stage to assign the TF family.

### 2.6 Evaluation and benchmarking protocol

To objectively evaluate our models and establish a fair baseline, we defined the benchmarking procedure described below. The test set contains 64 091 protein sequences from 58 families, representing a 20% stratified random sample from the original dataset. To provide a basis for comparison, we performed the same classification task using the Basic Local Alignment Search Tool, BLAST (Altschul *et al.* 1990). A BLAST reference database was generated using our training set, which contains 256 279 protein sequences (80% of the original dataset). For each entry in the test set, BLAST assigned the protein to the family with the highest similarity rate in the reference dataset and an identity threshold of 80%. This identity cutoff was selected to balance sensitivity and specificity, as lower thresholds (e.g. 50–60%) can introduce false positives by aligning distantly related sequences, while higher thresholds (e.g. 90–95%) may miss true homologs due to natural sequence divergence. An 80% identity level has been widely used in previous studies for family-level classification tasks, providing a robust compromise between accurate homology detection and generalization across diverse TF families. The ground truth labels in the database were used to evaluate both the BLAST results and our machine learning model. Based on these comparisons, we calculated various metrics, including accuracy, precision, recall, and *F*1-score, as described in Equations (1)–(4). For our proposed machine learning approach, we combined the predictions of the binary classifiers with two methods: a second-stage classifier (stacking) and a max-voting approach.

### 2.7 Evaluation metrics

We used a set of standard classification metrics to evaluate the performance of both the individual binary classifiers and the final classifier (via stacking or max-voting). These metrics provide a comprehensive overview of the model’s ability to correctly classify protein TF families. The following metrics were calculated:


(1) $$\text{Accuracy}=\frac{\text{TP}+\text{TN}}{\text{TP}+\text{TN}+\text{FP}+\text{FN}}$$

(2) $$\text{Precision}=\frac{\text{TP}}{\text{TP}+\text{FP}}$$

(3) $$\text{Recall}=\frac{\text{TP}}{\text{TP}+\text{FN}}$$

(4) $$F1\;\text{Score}=\frac{2\cdot \text{Precision}\cdot \text{Recall}}{\;\text{Precision}+\text{Recall}}$$

### 2.8 Implementation and availability of MegaPlantTF

We implemented all data processing, model inference, and visualization functionalities in a Python-based package named MegaPlantTF. This tool integrates the full MegaPlantTF workflow from *k*-mer feature extraction and ANOVA-based feature selection to binary model inference and result visualization within a reproducible software framework. The package provides a modular Python API to facilitate both simple use cases and large-scale analyses. The MegaPlantTF source code, along with example datasets and pretrained MegaPlantTF models, is freely available at: https://github.com/Bioinformatics-UM6P/MegaPlantTF MegaPlantTF can also be run directly on our webserver at: https://bioinformatics.um6p.ma/MegaPlantTF.

**Figure 3.**
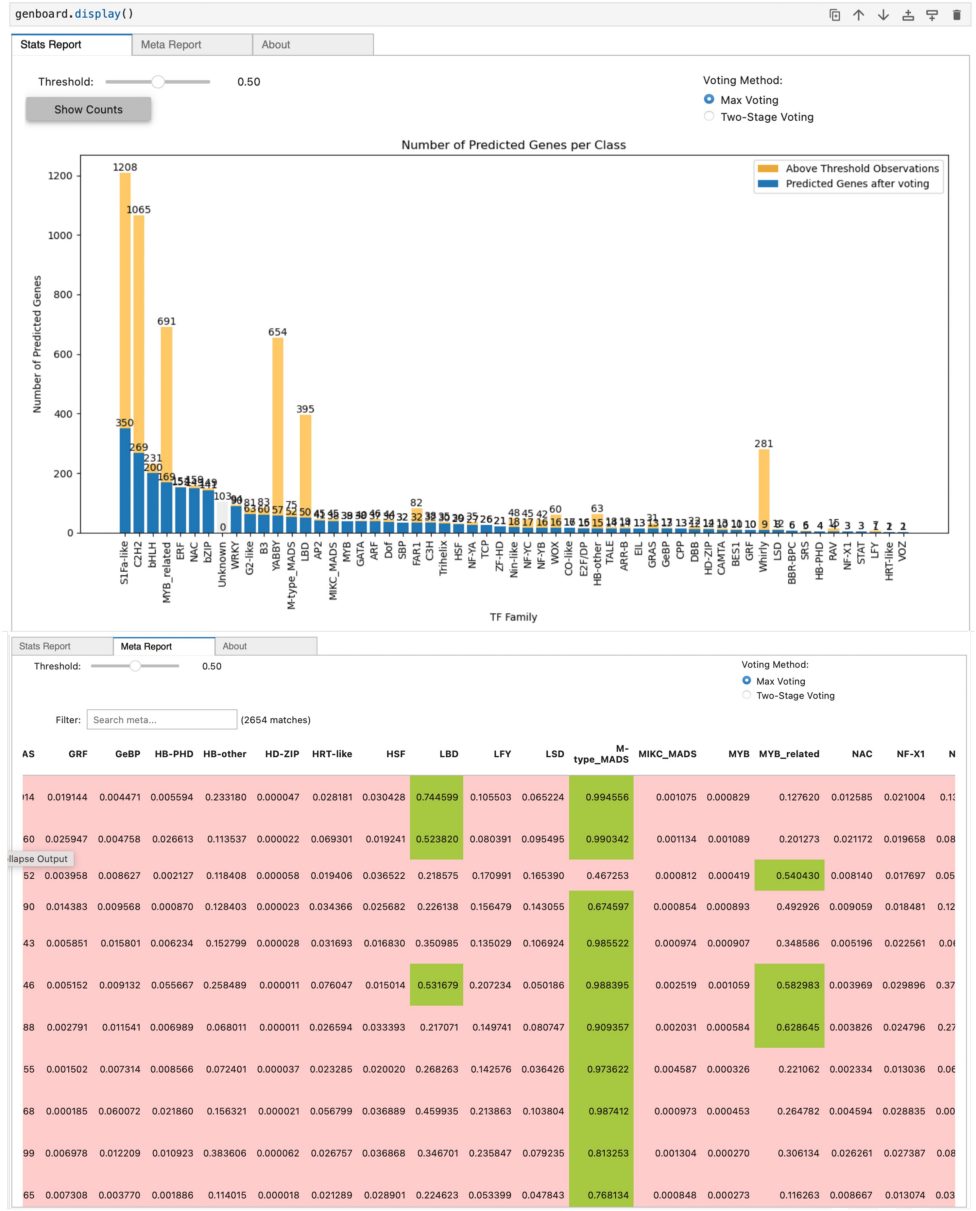
Overview of the post-processing interface for sequences classification. The post-processing interface presents a detailed classification report for each analyzed sequence. It displays the probability scores generated by all binary classifiers across transcription factor families. Cells highlighted in green indicate predictions that exceed the confidence threshold, signifying strong classifier support for that TF family assignment.

We have developed an inference class available in MegaPlantTF API that provides an interface for the efficient use of the trained models. First, users can provide the input as a fasta file and select the desired *k*-mer size for analysis. The inference module processes the file and generates the corresponding features based on the selected *k*-mer size. The system then automatically loads the appropriate pre-trained classifier along with the corresponding feature mask, ensuring that the model is tuned to the selected *k*-mer size. Once the features are processed, the module performs an inference with each classifier. The results are presented through an interface that allows the user to examine and analyze the predictions. In addition, the interface offers various voting methods for the final classification, including options such as max voting and a two-stage voting approach. The user has the option to set the confidence threshold, i.e. the minimum probability required for a class to be included in the final output. If no class meets this threshold, the sequence is classified as “unknown”, increasing the reliability and confidence in the classification results (see Fig. 3).

This interface, shown in Fig. 3, displays the number of predicted genes per TF family based on the selected voting method and threshold. The chart illustrates the number of genes assigned to each TF family after classification using the max-voting or the two-stage voting approach. The blue bars represent the genes that were assigned to a class after voting, while the orange bars show the genes that exceed the threshold for each family, giving an insight into the distribution and confidence of the predictions.

**Figure 4.**
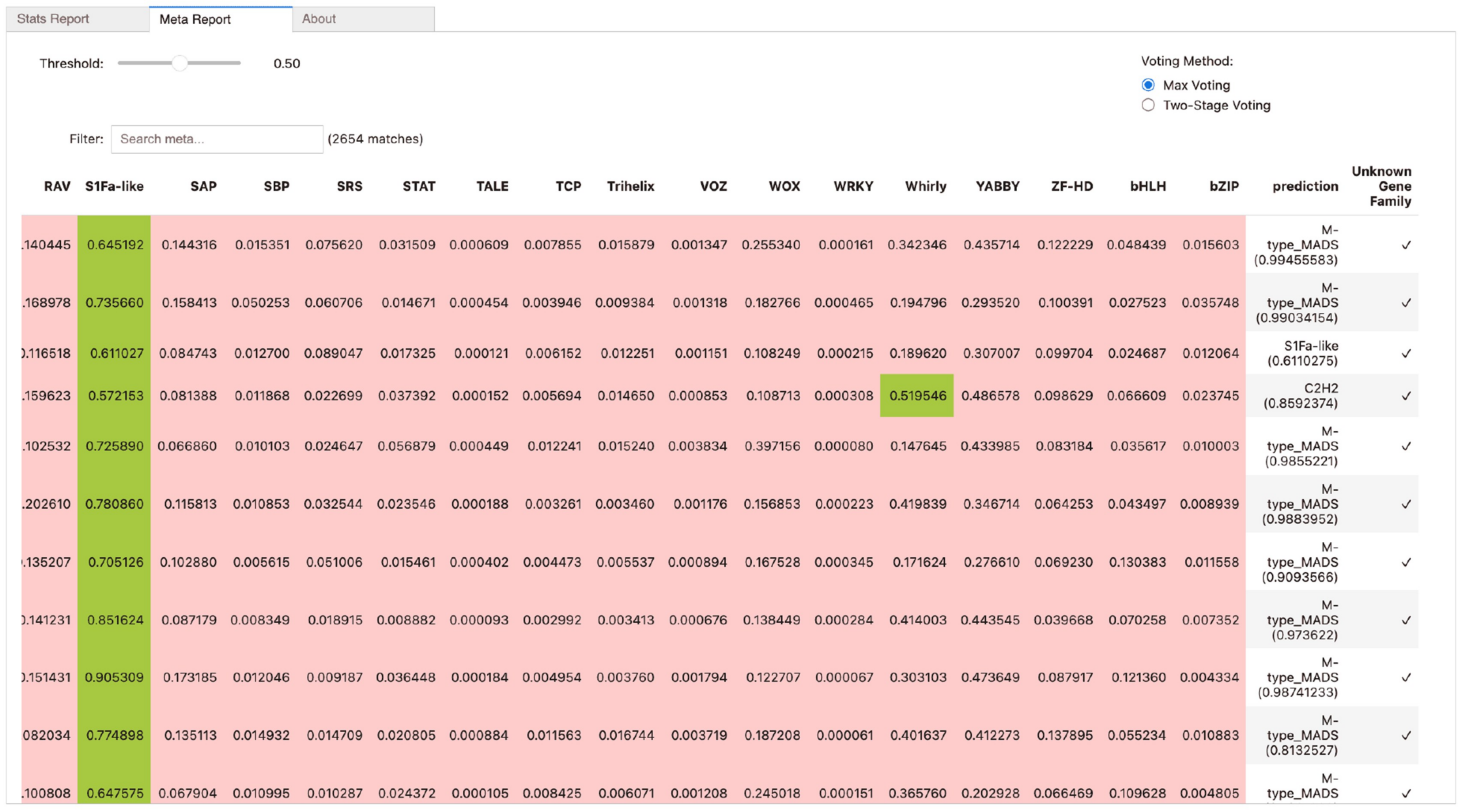
A detailed view of the final column showing the assigned TF family based on the selected threshold and voting method (max voting or two-stage voting).

Finally, the user interface includes a special tab with a detailed classification report that helps users to thoroughly analyze the results of the predictions. This report displays not only the probability scores generated by each binary classifier, but also the final assigned labels and confidence levels for each sequence in the input. By providing a detailed breakdown of classifier contributions and decision thresholds, this feature improves interpretability and assists users in evaluating the reliability and precision of predicted TF families (see Fig. 4).

## 3 Results

This section presents the results of our experiments, in which both training and testing phases were performed to evaluate the effectiveness of our approach in accurately classifying protein TF families. The evaluation was performed using standard performance metrics, while additional case studies were implemented to investigate the ability of the approach to classify TFs in more realistic and complex scenarios. These evaluations provide deeper insights into the generalizability and robustness of the model in practical applications. In addition to the standard cross-validation tests, we performed a case study on *Sorghum bicolor* to test the generalizability to unknown species and the performance of our method in a genome-wide study.

### 3.1 Performance comparison across classifier models

#### 3.1.1 Binary classifier performance

**Figure 5.**
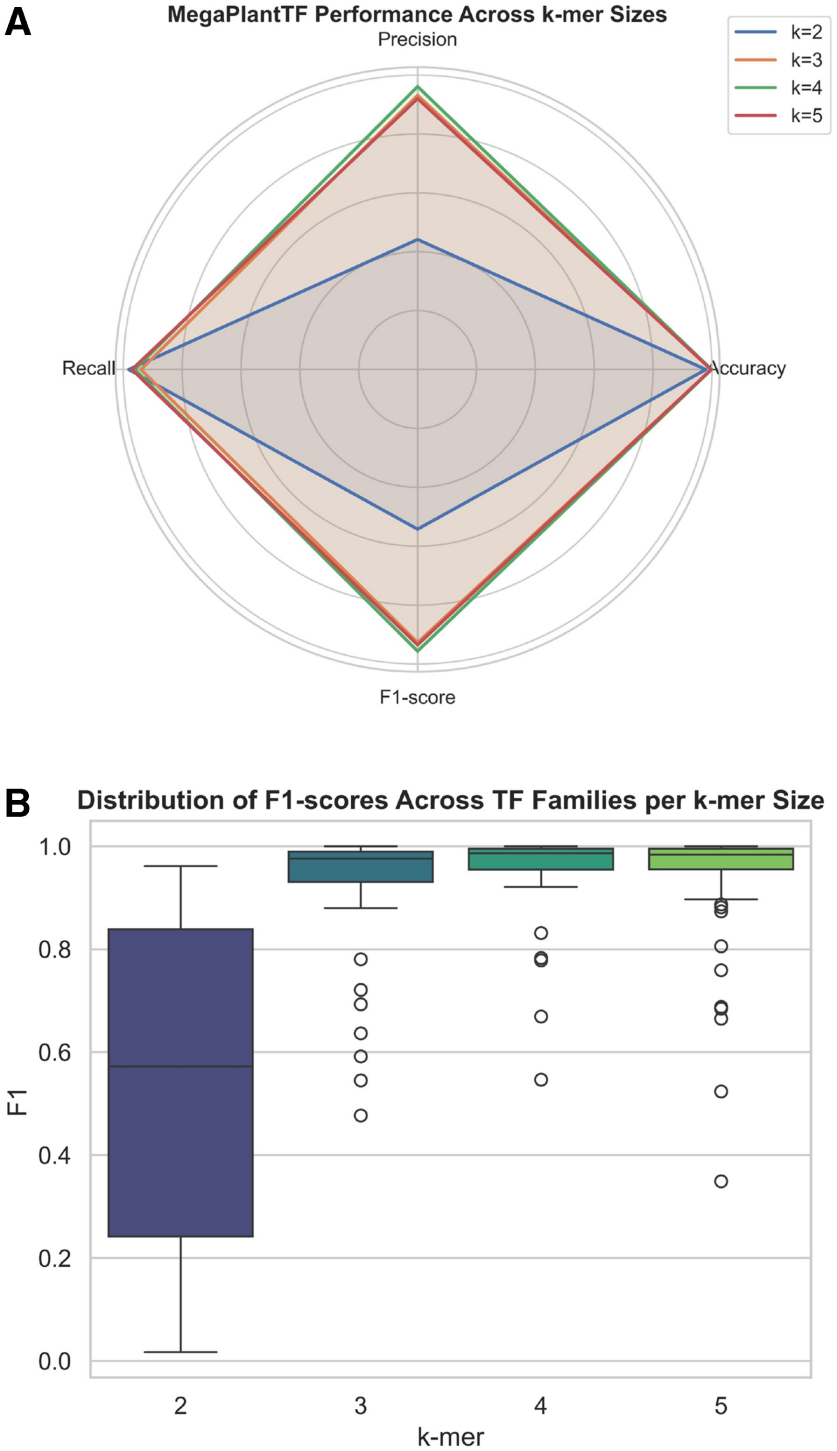
Comparison of MegaPlantTF performance across *k*-mer sizes. (A) Radar plot showing mean accuracy, precision, recall, and *F*1-score aggregated across all transcription factor (TF) families for each *k*-mer size. The model achieves consistently high performance for k=3 and k=4. (B) Boxplot illustrating the distribution of *F*1-scores across all TF families per *k*-mer size. The larger variance at k=2 indicates underrepresentation of sequence features, while k=3 and k=4 provide stable and robust classification performance.

The performance of the binary classifiers was evaluated using standard metrics, including accuracy, precision, recall, and *F*1-score, across different *k*-mer lengths (k=2,3,4,5). The detailed results are provided in Table S2, available as supplementary data at *Bioinformatics* online. As shown in Fig. 5, most classifiers demonstrate consistently high performance across *k*-mer sizes, with k=3, k=4, and k=5 yielding optimal results in terms of accuracy, precision, and *F*1-score. This robust pattern indicates that these configurations capture the most informative sequence features while maintaining computational efficiency. Consequently, k=3, k=4, and k=5 are considered the most effective *k*-mer sizes for building reliable foundational models suitable for integration into more complex, multi-stage classification pipelines. A detailed view of each binary classifier’s performance metrics is provided in Fig. 3, available as supplementary data at *Bioinformatics* online.

**Table 2.** Classification performance on Sorghum bicolor sequences for different threshold values. Metrics are calculated using the micro-averaged approach.^a^

	Generalizability to new species			Classification on whole genome data		
Threshold	Accuracy	Precision	Recall	Accuracy	Precision	Recall
0.5	**0.97**	**0.97**	**0.97**	0.32	0.32	0.32
0.95	0.89	0.89	0.89	0.78	0.78	0.78
0.98	0.83	0.83	0.83	**0.83**	**0.83**	**0.83**
0.99	0.76	0.76	0.76	**0.89**	**0.89**	**0.89**

a Experiment 1 involves testing on Sorghum sequences after removing *Sorghum* sequences from training. Experiment 2 involves testing *Sorghum’*s whole genome after excluding *Sorghum* sequences from training. **For each experiment, the best-performing results for accuracy, precision, and recall across all evaluated threshold values are highlighted in bold**.

#### 3.1.2 Final classifier evaluation (voting)

In addition to evaluating the individual binary classifiers, we employed two final classification methods—max voting and a second-stage stacking classifier—to combine the results of the binary classifiers for a refined prediction. The comparison between the final models and the traditional Basic Local Alignment Search Tool (BLAST) method is shown in Table 1, which presents the results for different *k*-mer sizes and classification thresholds. Three evaluation strategies were used to interpret the results:

- Micro-average metrics: These metrics were calculated globally for all protein sequences, taking into account the total number of true positives, false positives and false negatives. This approach emphasizes the overall predictive power of the model.
- Macro-average metrics: This method calculates the performance metrics for each TF family independently and then averages these values, treating all families equally regardless of their size. This is particularly useful for evaluating model performance for smaller or underrepresented TF families.
- Weighted-average metrics: This approach also calculates metrics for each family, but weights them by the number of sequences within each family. This is useful to understand how well the model performs on more frequent classes without ignoring less frequent classes.

#### 3.1.3 Threshold-based evaluation

We have introduced a probability threshold to increase classification confidence for both max voting and stacking classifiers. In max voting, the class with the highest probability is selected as the final prediction. By introducing thresholds of 0, 0.5, and 0.95, we have adjusted the stringency of the classification: A threshold of 0 selects the class with the highest probability without restriction, while higher thresholds (0.5, 0.95) require that the class with the highest probability exceeds a minimum confidence level before it is selected.

The results in Table 1 show that the second-stage stacking classifier outperforms both the max-voting and BLAST approaches, especially at a *k*-mer size of 3 and a threshold of 0.0. The stacking classifier achieved an impressive accuracy of **99%**, along with a precision score of **99%**. Even at the highest thresholds of 0.5 and 0.95, the method was able to maintain a robust classification by achieving **99%** and **98%** of the test set correctly. This shows that the stacking model is able to make very reliable and accurate predictions, making it a promising tool for protein classification tasks.

### 3.2 Statistical validation of model performance

To verify that the observed improvements of MegaPlantTF over BLAST are statistically significant, we performed a Mann–Whitney *U*-test on the per-family *accuracy* and *F1-scores* obtained from both methods. This non-parametric test was selected because it does not assume normality and is well suited for comparing independent performance distributions across TF families.

The results show that MegaPlantTF achieves significantly higher scores than BLAST across all evaluated metrics. Specifically, the Mann–Whitney *U*-statistic for accuracy was 3331.5 (*P* = 4.26 × 10^−20^) and for *F*1-score was 2987.5 (*P* = 2.82 × 10^−13^), both indicating strong statistical significance (*P* < .001). These results confirm that the observed improvements of MegaPlantTF are not attributable to random fluctuations but reflect a consistent and meaningful performance advantage over the BLAST baseline. The statistical validation thus reinforces the robustness and reliability of our proposed machine learning framework.

### 3.3 Cross-species performance and phylogenetic influence

**Figure 6.**
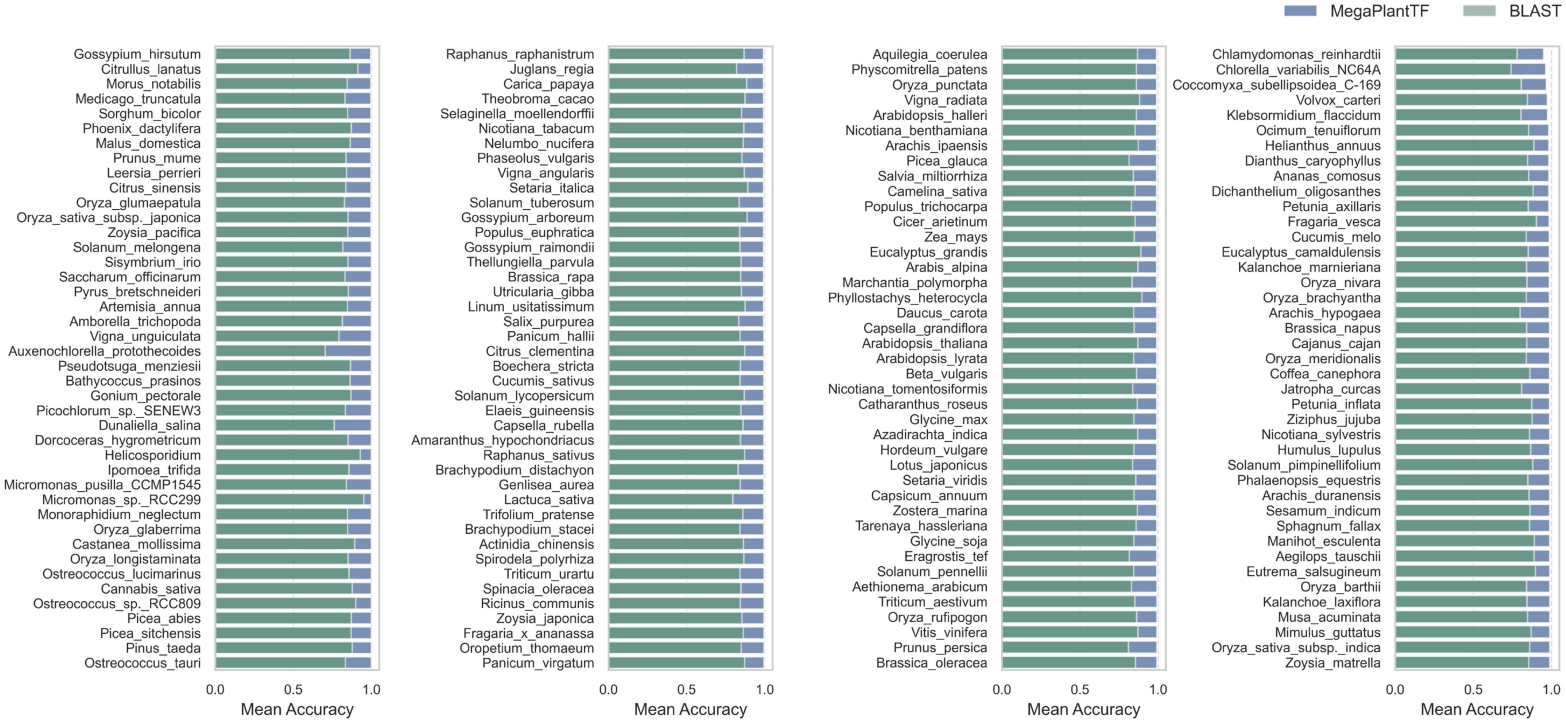
Cross-species comparison of MegaPlantTF and BLAST classification performance. Each bar represents the mean accuracy for a plant species, aggregated across all transcription factor families. MegaPlantTF consistently achieves higher or comparable accuracy to BLAST across diverse species, demonstrating strong cross-species generalization and limited phylogenetic bias.

To assess whether MegaPlantTF’s classification accuracy is affected by phylogenetic relationships among plant species, we evaluated model performance at the species level and compared it against a BLAST-based baseline. The objective was to determine whether MegaPlantTF achieves cross-lineage generalization rather than species-specific overfitting. Figure 6 summarizes the mean classification accuracy across all TF families for each species. MegaPlantTF consistently outperforms BLAST in nearly all cases, maintaining high accuracy across diverse taxa, including both model and non-model plants. This demonstrates that MegaPlantTF effectively captures conserved sequence features underlying TF domains, beyond simple sequence similarity. A complementary comparison based on the *F*1-score metric is provided in Fig. 4, available as supplementary data at *Bioinformatics* online, which likewise shows that MegaPlantTF achieves consistently superior performance to BLAST at the species level.

**Figure 7.**
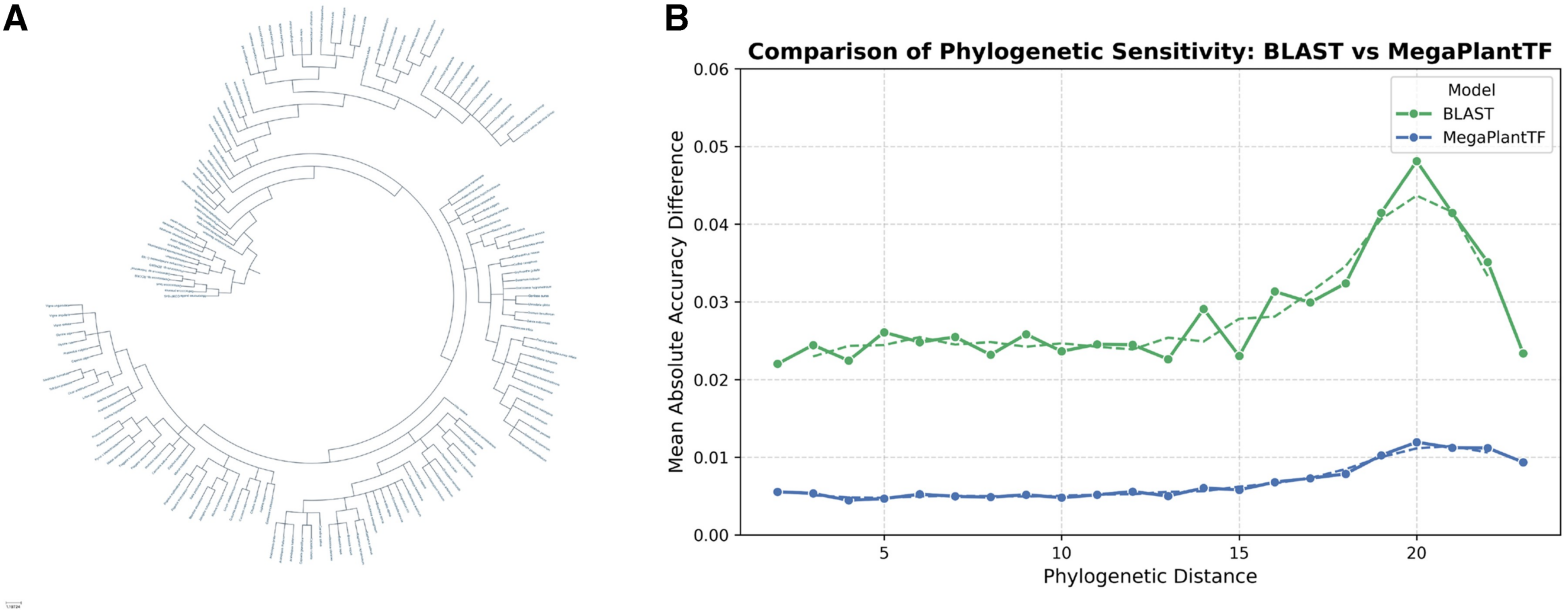
Phylogenetic context and sensitivity analysis of MegaPlantTF and BLAST performance. (A) Circular phylogenetic tree illustrating the evolutionary relationships among the evaluated plant species used for cross-species benchmarking. (B) Comparison of phylogenetic sensitivity between BLAST and MegaPlantTF, showing the mean absolute accuracy difference as a function of phylogenetic distance. MegaPlantTF maintains low sensitivity to phylogenetic divergence, demonstrating consistent performance across evolutionarily distant species, whereas BLAST accuracy decreases with increasing phylogenetic distance.

Figure 7 further explores the phylogenetic context and sensitivity of the models. Panel A shows the circular phylogenetic tree of the evaluated species, providing an evolutionary framework for the analysis. Panel B quantifies phylogenetic sensitivity by plotting the mean absolute accuracy difference as a function of phylogenetic distance. While BLAST performance declines with increasing divergence, MegaPlantTF remains stable, confirming its robustness and minimal dependency on evolutionary distance. Together, these results highlight MegaPlantTF’s strong generalization ability across plant lineages and its advantage over similarity-based prediction methods.

### 3.4 Case studies using the genome of *S. bicolor*

In this section, we evaluate the ability of the model to generalize to unseen species and its ability to distinguish TFs from other types of proteins. To evaluate these aspects, two specific case studies are performed:

#### 3.4.1 Generalization to new species

**Figure 8.**
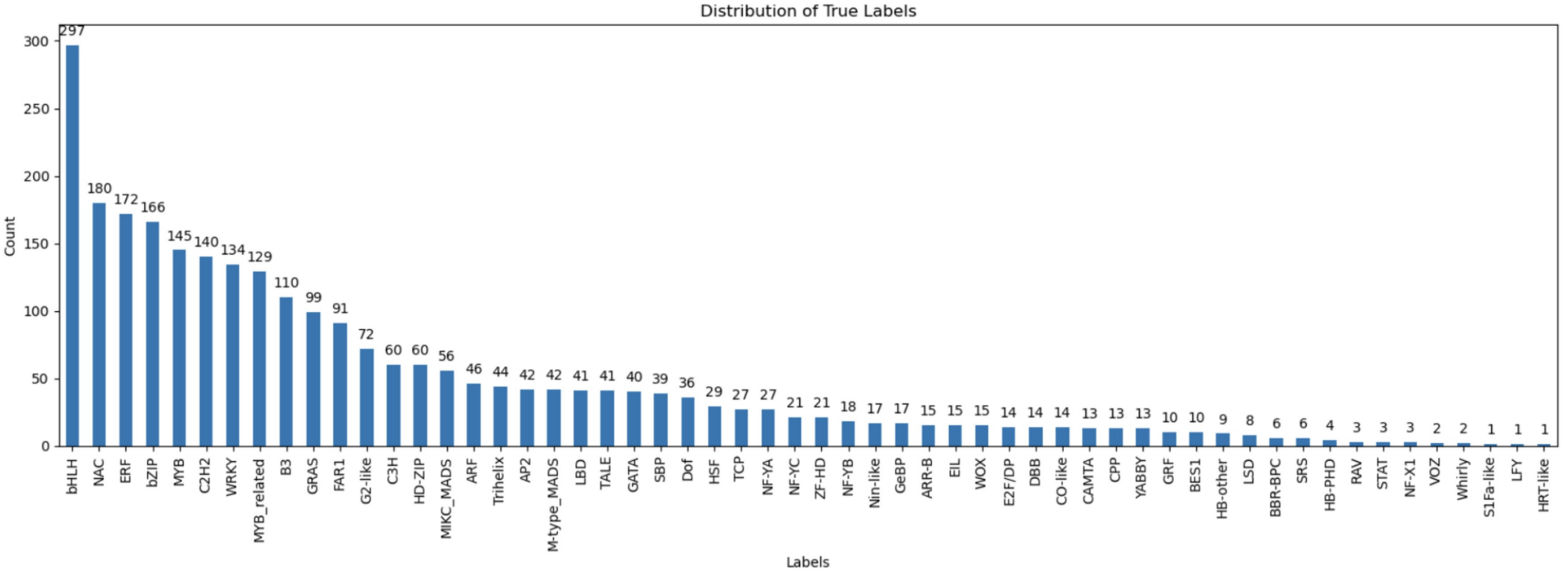
Distribution of transcription factor families in the *Sorghum bicolor* test set.

The first case study aims to determine whether the model can accurately predict TF families for protein sequences from species that were not included in the training set. Our initial training dataset includes protein sequences from 166 species. To test the generalization capabilities of the model, we removed 2654 sequences belonging to *S. bicolor* from the dataset, creating a scenario in which the species is completely unknown during training. Figure 8 describes the distribution of TF families in this test set.

Using the same pipeline already described in Section 2, we trained binary classifiers and second-stage classifiers and selected the best configuration from Table 1. For this experiment, we retrained the model with a *k*-mer size of 3 and applied a confidence threshold of 0.5. The retrained model was then evaluated on the protein sequences of *S. bicolor* to measure its generalization to this previously unseen species.

Table 2 shows the classification performance of the model for TFs of *S. bicolor*. This evaluation shows how well the model can adapt to new species. At a threshold of 0.5, the model performed well and achieved an accuracy of 0.97.

#### 3.4.2 Classification on whole genome data

**Figure 9.**
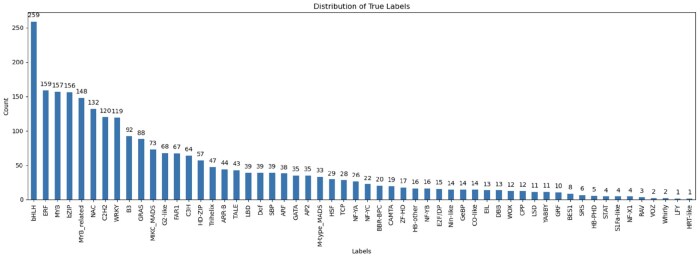
Distribution of transcription factors in the *Sorghum bicolor* whole genome test set.

In this second case study, we evaluated the performance of the model on a whole genome dataset to assess its ability to discriminate TFs from other protein families on a genome-wide scale. For this experiment, we processed the entire genome of *S. bicolor*, which contains both TFs and other protein sequences that cannot be assigned to any TF family. After obtaining the complete set of protein sequences from the NCBI database, we performed a BLAST analysis to re-annotate the sequences and categorize them into two classes: TFs and sequences without TFs. Figure 9 illustrates the distribution of the test set, which includes 39 248 protein sequences with 36 725 non-TF sequences.

We used the same binary classifiers and second-stage classifier configuration as in the previous case study (with *k* = 3) and ensured that all *S. bicolor* sequences were excluded from the training dataset. The results of this genome-wide analysis are summarized in Table 2. The highest precision achieved in this evaluation specifically for the non-TFs was 0.83, obtained with a confidence threshold of 0.98. This suggests that for mixed protein data, we should use the highest threshold—an adjustable parameter of the model—to confidently identify both TF families and non-TF sequences.

### 3.5 Usability and reproducibility of MegaPlantTF

To demonstrate usability, the MegaPlantTF tool allows users to reproduce our main analyses or predict TF families in their own proteomes with a single command. A typical workflow consists of three steps: (i) providing input sequences in FASTA format, (ii) selecting the desired *k*-mer size and threshold parameters, and (iii) executing inference using the pretrained MegaPlantTF models. The tool automatically handles feature generation, model loading, prediction, and post-processing, producing detailed classification reports and visual summaries similar to Figs 3 and 4. Example commands and datasets are provided in the online documentation to facilitate direct reproducibility. The current implementation of our work and the trained models and their weights are available at: https://bioinformatics.um6p.ma/MegaPlantTF/. Users who prefer to run MegaPlantTF online can use our webserver at: https://github.com/Bioinformatics-UM6P/MegaPlantTF. The webserver enables users to visualize the output directly on their browser, with the option to adjust the threshold and instantly view the corresponding results.

## 4 Discussion and conclusion

Various computational approaches or similarity-based techniques which are resource-intensive are used for predicting TF families from protein sequences. This is an essential step toward understanding gene regulatory mechanisms in different species. Our study introduces a robust classification framework based on a two-stage classification approach. In the first stage, we have a binary classifier for each TF family and aggregate the results of these classifiers using a second classifier in the second stage (stacking classifier). The analysis of the performance of the binary classifier (Fig. 5, Table 1 and Table 2, available as supplementary data at *Bioinformatics* online) shows that the *k*-mer size has a significant impact on the prediction accuracy of the model. Specifically, classifiers trained with a *k*-mer size of 3 performed consistently on various metrics, including accuracy, precision, recall, and *F*1-score. This suggests that a *k*-mer size of 3 strikes an optimal balance between capturing sequence features and maintaining computational efficiency. Interestingly, accuracy decreases at a *k*-mer size of 5, suggesting that larger *k*-mers may cause the model to memorize sequences rather than generalize effectively. This observation could also be relevant for other applications, such as data compression. In our evaluation of the final classifier, both the max-voting and stacking classifiers showed better performance compared to a baseline approach using BLAST. In particular, the stacking classifier achieved an accuracy of 0.994 and 0.980 with a threshold of 0.5 and 0.95, respectively, suggesting that it can effectively summarize information from multiple binary classifiers. The introduction of thresholds enabled a more nuanced approach to classification and increased confidence in the model’s predictions.

In addition to cross-validation, we investigated the model’s ability to generalize to new species and specifically evaluated its performance on protein sequences from a genome that was not included in the training dataset. Using the case study of the *S. bicolor* genome, the model maintained an accuracy of 0.97 when tested on this unseen species, demonstrating its robustness and adaptability. This ability is crucial in real-world applications where the model may encounter unannotated sequences or unknown species. We also evaluated the performance of the model on a genome-wide scale, highlighting its effectiveness in discriminating TFs from whole genome protein sequences. The results show that the model can achieve an accuracy of 0.83 and 0.89 with a threshold of 0.98 and 0.99, respectively. This emphasizes the idea that using higher thresholds is beneficial when dealing with mixed protein data and ensures a more confident classification of both TFs and non-TFs. Since the user can flexibly choose the threshold value, MegaPlantTF can be used for both TF classification and whole genome-level protein classification.

Furthermore, another promising research direction involves replacing or augmenting *k*-mer–based encoding with pretrained protein embeddings derived from large-scale sequence language models. Similar to genomic language modeling approaches explored in previous work (Akotenou and El Allali 2025), embedding-based architectures could enable the capture of deeper structural and contextual information within TF sequences. However, these methods are typically GPU-intensive and computationally demanding, which may limit their scalability in plant genomics. Nonetheless, incorporating pretrained embedding representations could serve as an alternative modeling paradigm to further enhance MegaPlantTF’s generalization capability across distant evolutionary lineages as well as whole genome scale TF identification.

In summary, our study shows that the proposed classification framework is not only able to accurately classify TF families from different datasets, but also exhibits robust generalizability to unknown species and whole genome sequences. The combination of deep learning-based binary classifiers and advanced ensemble techniques, such as stacking, improves the prediction performance and provides a reliable tool for protein classification tasks. Future work could explore the extension of this framework to other protein families and the integration of additional data modalities to further improve prediction accuracy.

Key points

- MegaPlantTF framework is a two-stage machine learning model combining deep learning and ensemble techniques to classify plant transcription factors (TFs) with improved accuracy and efficiency.
- The proposed stacking classifier achieved high precision (99%) and robustness outperforming traditional methods.
- MegaPlantTF demonstrated strong generalization in case studies, effectively classifying TFs in species excluded from the training set.
- The approach successfully distinguished transcription factors from other protein families on a Genome-Wide scale, highlighting its utility for large-scale genomic studies.

## Supplementary Material

btaf678_Supplementary_Data

## Data Availability

The current implementation of our work and the trained models and their weights are available at: github.com/Bioinformatics-UM6P/MegaPlantTF. MegaPlantTF online web server can also be accessed directly through the following URL: https://bioinformatics.um6p.ma/MegaPlantTF.

## Footnotes

1 (The final feature space size is chosen heuristically to optimize model performance and computational efficiency)
