# Supplementary material for: MegaPlantTF: a machine learning framework for comprehensive identification and classification of plant transcription factors
Source: Bioinformatics. 2025 Dec 23;42(1):btaf678. doi: 10.1093/bioinformatics/btaf678 (PMC12803907; doi:10.1093/bioinformatics/btaf678)
Supplement: btaf678_Supplementary_Data [file btaf678_supplementary_data.pdf]

Supplementary Materials

MegaPlantTF: A Machine Learning Framework for Comprehensive Identification and Classification of Plant Transcription Factors

Genereux Akotenou, Asmaa H. Hassan, Morad M. Mokhtar, Achraf El Allali*

Bioinformatics Laboratory, College of Computing, Mohammed VI Polytechnic University, Lot 660, Hay Moulay Rachid, Ben Guerir 43150, Morocco

Contents

S1.Protein class distribution in plant genomes from PlantTFDB 4.0 . . . . **2**

S2.Performance metrics for MegaPlantTF different binary-classifiers **3**

S3.Performance Comparison Across TF Binary Families **5**

S4.Cross-Species and Phylogenetic Generalization Analysis **6**

*Corresponding author: achraf.elallali@um6p.ma

S1. Protein class distribution in plant genomes from PlantTFDB 4.0

AP2 (4461)	ARF (4578)	ARR-B (2354)	B3 (10609)	BBR-BPC (1256)
BES1 (1549)	C2H2 (17740)	C3H (9693)	CAMTA (1343)	CO-like (2125)
CPP (1612)	DBB (1651)	Dof (5655)	E2F/DP (1781)	EIL (1234)
ERF (21129)	FAR1 (7527)	G2-like (9874)	GATA (5335)	GRAS (9304)
GRF (1876)	GeBP (1564)	HB-PHD (477)	HB-other (2277)	HD-ZIP (8602)
HRT-like (249)	HSF (4574)	LBD (7216)	LFY (253)	LSD (957)
Mt-type_MADS (7541)	MIKC_MADS (6918)	MYB (22032)	MYB_related (15369)	NAC (19997)
NF-X1 (403)	NF-YA (2461)	NF-YB (3099)	NF-YC (2446)	NZZ/SPL (109)
Nin-like (2766)	RAV (690)	S1Fa-like (359)	SAP (164)	SBP (4168)
SRS (1327)	STAT (214)	TALE (4433)	TCP (4187)	Trihelix (6256)
VOZ (635)	WOX (2358)	WRKY (14549)	Whirly (530)	YABBY (1719)
ZF-HD (2589)	bHLH (28698)	bZIP (15498)		

Supplementary Table S1: Protein class distribution in plant genomes from PlantTFDB 4.0

S2. Performance metrics for MegaPlantTF different binary-classifiers

Gene Family	Accuracy				Precision				Recall				F1			
	k=2	k=3	k=4	k=5	k=2	k=3	k=4	k=5	k=2	k=3	k=4	k=5	k=2	k=3	k=4	k=5
AP2	0.973	0.979	**0.985**	0.969	0.978	0.974	0.985	**0.987**	0.967	0.983	**0.984**	0.950	0.973	0.979	**0.985**	0.968
ARF	0.996	0.998	**0.999**	0.998	0.992	**1.000**	1.000	1.000	**1.000**	0.996	0.998	0.997	0.996	0.998	**0.999**	0.998
ARR-B	0.982	0.987	**0.988**	0.987	**1.000**	1.000	0.991	0.991	0.964	0.975	**0.985**	0.983	0.982	0.987	**0.988**	0.987
B3	**0.989**	0.959	0.984	0.932	0.995	**0.998**	0.998	0.998	**0.983**	0.919	0.969	0.865	**0.989**	0.957	0.983	0.927
BBR-BPC	0.988	0.996	**1.000**	0.992	0.988	**1.000**	1.000	1.000	0.988	0.992	**1.000**	0.984	0.988	0.996	**1.000**	0.992
BES1	0.987	**1.000**	1.000	0.997	0.981	**1.000**	1.000	1.000	0.994	**1.000**	1.000	0.994	0.987	**1.000**	1.000	0.997
C2H2	**0.976**	0.901	0.971	0.920	0.986	0.998	0.999	**1.000**	**0.966**	0.803	0.944	0.841	**0.976**	0.890	0.971	0.914
C3H	0.972	0.898	**0.987**	0.938	0.982	**1.000**	0.999	1.000	0.962	0.797	**0.974**	0.875	0.972	0.887	**0.986**	0.933
CAMTA	0.989	0.994	0.998	**1.000**	0.982	**1.000**	1.000	1.000	0.996	0.989	0.996	**1.000**	0.989	0.994	0.998	**1.000**
CO-like	0.993	0.995	**0.998**	0.998	0.998	**1.000**	1.000	1.000	0.988	0.991	**0.995**	0.995	0.993	0.995	**0.998**	0.998
CPP	0.988	0.995	**0.997**	0.986	0.988	0.997	**1.000**	1.000	0.988	**0.994**	0.994	0.972	0.988	0.995	**0.997**	0.986
DBB	0.970	0.715	**0.995**	0.991	0.953	**1.000**	0.994	0.997	0.988	0.429	**0.997**	0.985	0.970	0.600	**0.995**	0.991
Dof	0.993	0.997	**0.999**	0.998	0.996	**1.000**	1.000	1.000	0.989	0.995	**0.997**	0.996	0.993	0.997	**0.999**	0.998
E2F/DP	0.985	0.992	**1.000**	0.997	0.994	**1.000**	1.000	1.000	0.975	0.983	**1.000**	0.994	0.984	0.992	**1.000**	0.997
EIL	0.984	0.856	**0.992**	0.992	0.984	**1.000**	1.000	1.000	**0.984**	0.713	0.984	0.984	0.984	0.832	**0.992**	0.992
ERF	0.989	**0.997**	0.995	0.996	0.993	**0.998**	0.995	0.997	0.985	**0.996**	0.996	0.996	0.989	**0.997**	0.995	0.996
FAR1	0.981	**0.991**	0.987	0.967	0.983	0.998	**0.999**	0.999	0.979	**0.984**	0.975	0.936	0.981	**0.991**	0.987	0.966
G2-like	0.981	0.986	**0.993**	0.990	0.989	**0.998**	0.998	0.993	0.973	0.974	**0.988**	0.987	0.981	0.986	**0.993**	0.990
GATA	0.986	0.989	**0.998**	0.992	0.989	**1.000**	1.000	1.000	0.983	0.978	**0.996**	0.984	0.986	0.989	**0.998**	0.992
GRAS	0.986	0.985	**0.993**	0.989	0.995	**1.000**	0.999	1.000	0.977	0.970	**0.987**	0.978	0.986	0.985	**0.993**	0.989
GRF	0.997	0.997	**1.000**	0.999	0.997	**1.000**	1.000	1.000	0.997	0.995	**1.000**	0.997	0.997	0.997	**1.000**	0.999
GeBP	0.966	0.970	**0.981**	0.981	0.974	**1.000**	1.000	1.000	0.958	0.939	**0.962**	0.962	0.966	0.969	**0.980**	0.980
HB-PHD	**1.000**	0.995	0.995	1.000	**1.000**	1.000	1.000	1.000	**1.000**	0.990	0.990	1.000	**1.000**	0.995	0.995	1.000
HB-other	**0.944**	0.827	0.935	0.887	0.979	**0.987**	0.983	0.971	**0.908**	0.664	0.886	0.798	**0.942**	0.794	0.932	0.876
HD-ZIP	0.992	0.997	**0.998**	0.998	0.993	0.999	**1.000**	0.999	0.991	0.994	0.996	**0.998**	0.992	0.997	**0.998**	0.998
HRT-like	0.940	**1.000**	0.990	1.000	0.923	**1.000**	1.000	1.000	0.960	**1.000**	0.980	1.000	0.941	**1.000**	0.990	1.000
HSF	0.989	**0.999**	0.999	0.998	0.988	**1.000**	1.000	1.000	0.990	0.998	**0.999**	0.997	0.989	**0.999**	0.999	0.998
LBD	0.975	0.991	**0.995**	0.989	0.972	0.999	**1.000**	0.999	0.979	0.982	**0.990**	0.979	0.975	0.991	**0.995**	0.989
LSD	0.979	0.971	0.997	**1.000**	0.969	**1.000**	1.000	1.000	0.990	0.943	0.995	**1.000**	0.979	0.971	0.997	**1.000**

Supplementary Table S2: Performance metrics for different binary-classifiers. The light-blue and bold numbers indicate the maximum values in their respective rows given each evaluation metric (1).

Gene Family	Accuracy				Precision				Recall				F1			
	k=2	k=3	k=4	k=5	k=2	k=3	k=4	k=5	k=2	k=3	k=4	k=5	k=2	k=3	k=4	k=5
LFY	0.941	0.931	**0.990**	0.971	0.925	**1.000**	1.000	1.000	0.961	0.863	**0.980**	0.941	0.942	0.926	**0.990**	0.970
M-type_MADS	0.953	0.952	**0.985**	0.984	0.952	**0.998**	0.985	0.997	0.953	0.906	**0.985**	0.972	0.953	0.950	**0.985**	0.984
MIKC_MADS	0.994	0.993	0.995	**0.996**	0.993	**0.999**	0.994	0.995	0.996	0.986	0.996	**0.997**	0.994	0.993	0.995	**0.996**
MYB	0.989	0.944	**0.993**	0.990	0.985	**0.998**	0.997	0.996	**0.993**	0.889	0.988	0.984	0.989	0.940	**0.993**	0.990
MYB_related	0.915	0.842	**0.937**	0.924	0.901	**0.991**	0.977	0.979	**0.932**	0.691	0.896	0.866	0.916	0.814	**0.935**	0.919
NAC	0.989	0.986	**0.994**	0.979	0.996	**1.000**	0.999	1.000	0.981	0.972	**0.988**	0.958	0.989	0.986	**0.994**	0.979
NF-X1	0.994	**1.000**	1.000	0.988	0.988	**1.000**	1.000	1.000	**1.000**	1.000	1.000	0.975	0.994	**1.000**	1.000	0.988
NF-YA	0.992	0.998	**0.999**	0.998	0.992	**1.000**	1.000	1.000	0.992	0.996	**0.998**	0.996	0.992	0.998	**0.999**	0.998
NF-YB	0.973	0.996	**0.998**	0.997	0.971	**1.000**	1.000	1.000	0.974	0.992	**0.995**	0.994	0.973	0.996	**0.998**	0.997
NF-YC	0.965	0.944	**0.996**	0.979	0.949	**1.000**	1.000	1.000	0.984	0.888	**0.992**	0.957	0.966	0.941	**0.996**	0.978
NZZ/SPL	**1.000**	1.000	0.977	0.977	**1.000**	1.000	1.000	1.000	**1.000**	1.000	0.955	0.955	**1.000**	1.000	0.977	0.977
Nin-like	**0.995**	0.973	0.986	0.977	0.998	**1.000**	1.000	1.000	**0.993**	0.946	0.971	0.955	**0.995**	0.972	0.985	0.977
RAV	0.993	0.989	0.996	**1.000**	0.986	0.993	**1.000**	1.000	**1.000**	0.986	0.993	1.000	0.993	0.989	0.996	**1.000**
S1Fa-like	0.972	**0.993**	0.979	0.979	0.947	**1.000**	1.000	1.000	**1.000**	0.986	0.958	0.958	0.973	**0.993**	0.979	0.979
SAP	**1.000**	1.000	1.000	0.985	**1.000**	1.000	1.000	1.000	**1.000**	1.000	1.000	0.970	**1.000**	1.000	1.000	0.985
SBP	0.968	0.971	**0.998**	0.992	0.960	**1.000**	1.000	1.000	0.976	0.942	**0.995**	0.984	0.968	0.970	**0.998**	0.992
SRS	0.989	0.964	0.998	**1.000**	0.989	**1.000**	1.000	1.000	0.989	0.929	0.996	**1.000**	0.989	0.963	0.998	**1.000**
STAT	**1.000**	1.000	1.000	1.000	**1.000**	1.000	1.000	1.000	**1.000**	1.000	1.000	1.000	**1.000**	1.000	1.000	1.000
TALE	0.992	0.999	**1.000**	1.000	0.993	0.999	**1.000**	1.000	0.990	**1.000**	1.000	1.000	0.992	0.999	**1.000**	1.000
TCP	0.982	0.996	**0.998**	0.995	0.982	**1.000**	1.000	1.000	0.982	0.993	**0.996**	0.990	0.982	0.996	**0.998**	0.995
Trihelix	0.981	0.950	**0.990**	0.971	0.989	**1.000**	1.000	1.000	0.974	0.900	**0.979**	0.942	0.981	0.947	**0.990**	0.970
VOZ	0.992	**1.000**	1.000	0.996	0.992	**1.000**	1.000	1.000	0.992	**1.000**	1.000	0.992	0.992	**1.000**	1.000	0.996
WOX	0.975	**0.998**	0.998	0.998	0.965	**1.000**	1.000	1.000	0.985	**0.996**	0.996	0.996	0.975	**0.998**	0.998	0.998
WRKY	0.991	0.993	**0.998**	0.997	0.995	**1.000**	1.000	1.000	0.986	0.987	**0.996**	0.995	0.991	0.993	**0.998**	0.997
Whirly	0.943	**1.000**	1.000	0.995	0.912	**1.000**	1.000	1.000	0.981	**1.000**	1.000	0.991	0.945	**1.000**	1.000	0.995
YABBY	0.977	**0.999**	0.990	0.991	0.961	**1.000**	1.000	1.000	0.994	**0.997**	0.980	0.983	0.977	**0.999**	0.990	0.991
ZF-HD	0.983	**0.997**	0.997	0.996	0.998	**1.000**	1.000	1.000	0.967	**0.994**	0.994	0.992	0.982	**0.997**	0.997	0.996
bHLH	**0.983**	0.925	0.983	0.979	0.987	**1.000**	0.999	0.999	**0.978**	0.850	0.967	0.959	**0.983**	0.919	0.983	0.979
bZIP	0.986	0.942	**0.994**	0.990	0.996	**1.000**	0.999	1.000	0.975	0.885	**0.989**	0.979	0.986	0.939	**0.994**	0.989

Supplementary Table S3: Performance metrics for different binary-classifiers. The light-blue and bold numbers indicate the maximum values in their respective rows given each evaluation metric (2).

S3. Performance Comparison Across TF Binary Families

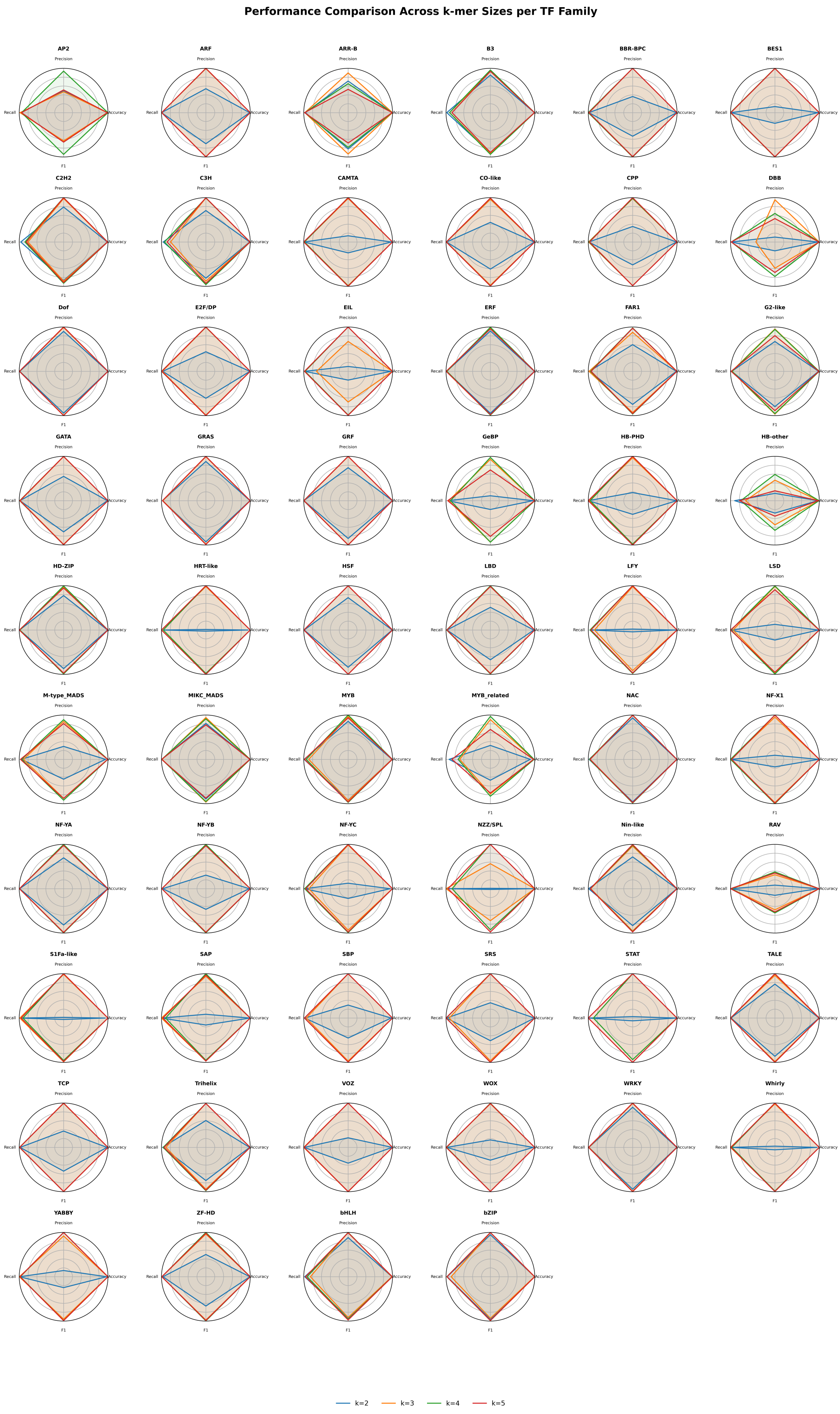

Supplementary Figure S1: Performance comparison across k-mer sizes for each transcription factor binary family. Each radar plot summarizes the performance metrics—accuracy, precision, recall, and F1-score—of the corresponding binary classifier trained with $k = 2$, $k = 3$, $k = 4$, and $k = 5$.

S4. Cross-Species and Phylogenetic Generalization Analysis

To evaluate the cross-species generalization and phylogenetic robustness of MegaPlantTF, we analyzed its classification performance across multiple plant genomes and compared it with BLAST. Figure S2 summarizes the results of this analysis.

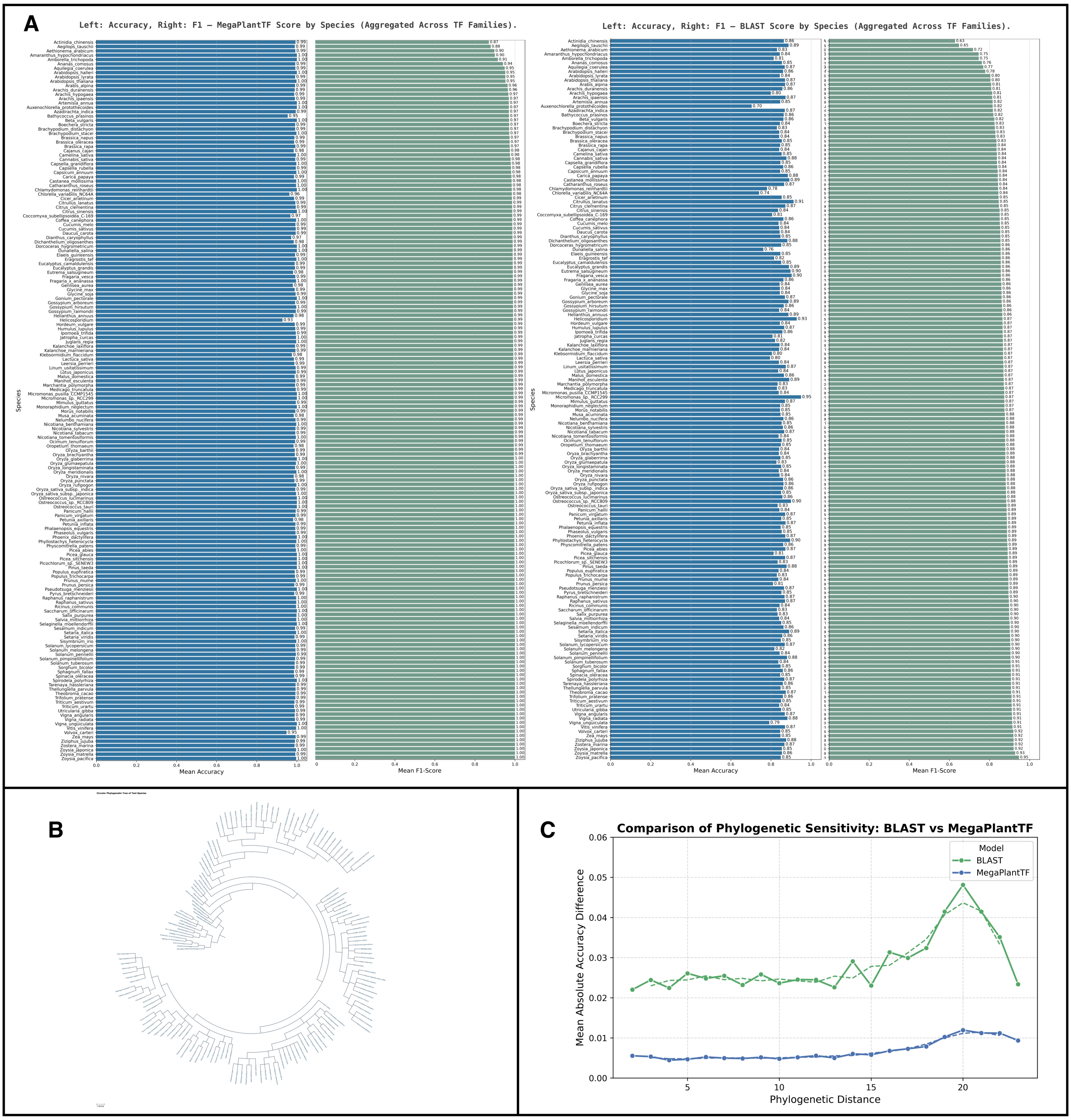

Supplementary Figure S2: Cross-species and phylogenetic comparison of MegaPlantTF and BLAST performance. **(A)** Mean accuracy and F1-score per species, aggregated across transcription factor (TF) families. **(B)** Circular phylogenetic tree showing evolutionary relationships among the evaluated species. **(C)** Phylogenetic sensitivity analysis displaying the mean absolute accuracy difference as a function of phylogenetic distance.
